# Antimicrobial Metabolites of Caucasian Medicinal Plants as Alternatives to Antibiotics

**DOI:** 10.3390/antibiotics13060487

**Published:** 2024-05-24

**Authors:** Marta Fik-Jaskółka, Valentina Mittova, Catherine Motsonelidze, Malkhaz Vakhania, Caterina Vicidomini, Giovanni N. Roviello

**Affiliations:** 1 Faculty of Chemistry, Adam Mickiewicz University in Poznań, Uniwersytetu Poznańskiego 8, 61-614 Poznań, Poland; 2 Teaching University Geomedi, 4 King Solomon II Str., Tbilisi 0114, Georgia; valentina.mittova@geomedi.edu.ge (V.M.);; 3 Institute of Biostructures and Bioimaging, Italian National Council for Research (IBB-CNR), Area di Ricerca Site and Headquarters, Via Pietro Castellino 111, 80131 Naples, Italy

**Keywords:** antimicrobial metabolites, antibiotic resistance, alternative treatments, pharmacological properties, Caucasian medicinal plants, phenolic compounds, flavonoids, tannins, terpenes, saponins, alkaloids, sulfur-containing compounds

## Abstract

This review explores the potential of antimicrobial metabolites derived from Caucasian medicinal plants as alternatives to conventional antibiotics. With the rise of antibiotic resistance posing a global health threat, there is a pressing need to investigate alternative sources of antimicrobial agents. Caucasian medicinal plants have traditionally been used for their therapeutic properties, and recent research has highlighted their potential as sources of antimicrobial compounds. Representatives of 15 families of Caucasian medicinal plant extracts (24 species) have been explored for their efficacy against these pathogens. The effect of these plants on Gram-positive and Gram-negative bacteria and fungi is discussed in this paper. By harnessing the bioactive metabolites present in these plants, this study aims to contribute to the development of new antimicrobial treatments that can effectively combat bacterial infections while minimizing the risk of resistance emergence. Herein we discuss the following classes of bioactive compounds exhibiting antimicrobial activity: phenolic compounds, flavonoids, tannins, terpenes, saponins, alkaloids, and sulfur-containing compounds of Allium species. The review discusses the pharmacological properties of selected Caucasian medicinal plants, the extraction and characterization of these antimicrobial metabolites, the mechanisms of action of antibacterial and antifungal plant compounds, and their potential applications in clinical settings. Additionally, challenges and future directions in the research of antimicrobial metabolites from Caucasian medicinal plants are addressed.

## 1. Introduction

A reduction in the number of infection-related deaths is a crucial worldwide public health objective, with the World Health Organization (WHO) ranking antibiotic resistance as one of the top 10 dangers to global public health [1]. In 2019, out of 13.7 million infection-related deaths, 7.7 million deaths were associated with 33 bacterial pathogens [2]. Antibiotic resistance [3,4] has led to a decreased effectiveness or even the complete ineffectiveness of current antibacterial medications [5]. Consequently, there is a growing demand to create novel antimicrobial drugs that can combat the emergence of resistance and reduce the need for antibiotics [1]. Various bioactive chemicals can be obtained from medicinal plants [6,7,8,9,10], and numerous techniques have been employed to investigate their potential as antimicrobial agents [11]. These bioactive compounds are secondary plant metabolites, representing one of the unexplored sources of antimicrobial agents in nature [12]. The results of phytochemical studies demonstrated that plant extracts may inhibit the growth of bacteria, fungi, viruses, and protozoa by mechanisms different from antibiotics and therefore may have significant clinical value in the treatment of resistant microbial strains [13]. Information regarding the traditional use of medicinal plants can be a useful basis for the identification of plants exhibiting pharmacological activity [14]. This is particularly true for biodiversity-rich regions of the world such as the Caucasus, with a history of long-time traditional (ethnobotanical) use of medicinal plants [15].

**Figure 1:**
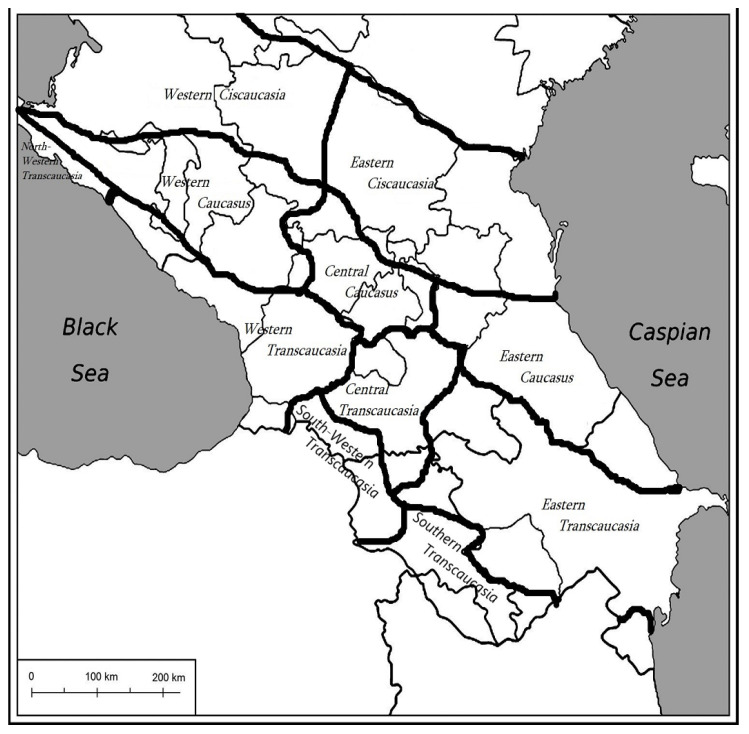
The borders and floristic districts of the Caucasus according to [19].

As a geographic, historical, and cultural phenomenon, the Caucasus covers a vast area between the Black Sea to the west and the Caspian Sea to the east [16]. In this study, we determined the floristic boundaries of the Caucasus following the guidelines outlined in [17]. The demarcation of primary natural zones within the Caucasus territory is established through floristic zoning, employing the “sectoral” principle as developed by A. A. Grossheim [18] and A. L. Takhtajan [19] (Figure 1). According to this principle, the territory of the Caucasus is divided into zones associated with the division of territory by Greater Caucasus and Lesser Caucasus mountain ranges [19]. These mountain systems and long-term climate changes, specifically the gradual decline in temperature and humidity, have contributed to the development of the Caucasus biodiversity hotspot [20,21]. In total, 6350 vascular plant species occur in the Caucasus, including more than 2900 endemic species [21]. Among these plant materials, 1000 species have therapeutic uses in traditional folk medicine and approximately 180 species are used in modern medicine [22]. The distribution of the Caucasian medicinal plant species in Table 1 is shown according to the floristic zones.

In this review, we conducted a systematic classification of Caucasian plants, focusing on their revealed antimicrobial properties, while also outlining their species range following the sectoral principle as described previously. Our literature search adhered to the Prisma guidelines for systematic reviews [67]. The literature search was carried out in PubMed, Scopus, ScienceDirect, and Google Scholar databases. A total of 207 publications were collected and assessed. The following search criteria were used: (1) “Antimicrobial”; (2) “Caucasian plants”; (3) “Caucasian Medicinal Plants”; (4) the name of each Caucasian medicinal plant, for which antimicrobial activity was demonstrated, was also used for the further search aimed at the identification of bioactive compounds, described for the particular species. All reported plant names were cross-checked following The Plant List (http://www.theplantlist.org/, accessed on 9 May 2024) and the International Plant Names Index (https://www.ipni.org/, accessed on 9 May 2024). Plant families were assigned using the Angiosperm Phylogeny Group IV guidance [68]. The names of all bacterial species discussed in the review were cross-checked with the LPSN (List of Prokaryotic Names) at https://www.bacterio.net/ (accessed on 9 May 2024); the names of fungi were cross-checked at https://www.indexfungorum.org/names/Names.asp (accessed on 9 May 2024).

## 2. Antimicrobial and Antifungal Activity of Caucasian Medicinal Plants

Throughout human history, different plant parts of medicinal plants such as leaves, stems, bark, roots, seeds, and fruits have been used for the treatment and prevention of all main types of diseases [69]. Biologically active compounds isolated from plant material still serve as the major sources of new drug molecules today. However, the revision of the published articles devoted to the antimicrobial effect of these biologically active compounds revealed that a comparison between results is often difficult due to the use of different non-standardized approaches. Various laboratory methods can be used for the in vitro assessment of the antimicrobial activity of a given extract or a pure compound [70]. These methods include diffusion methods (such as the agar disk diffusion method, the antimicrobial gradient method, the agar well diffusion method, the agar plug diffusion method, and the cross streak method), thin-layer chromatography (TLC)–bioautography methods (agar diffusion, direct bioautography), and dilution methods (broth dilution method, agar dilution method, time-kill test) [70]. The most common indicators of antimicrobial activity, measured by various methods, are the minimum inhibitory concentration (MIC) and zone of inhibition (IZ). The MIC is the lowest concentration of an antibacterial agent expressed in mg/L which, under strictly controlled in vitro conditions, completely prevents visible growth of the test strain of an organism [71]. Another indicator used in the study of antimicrobial activity is the zone of inhibition (IZ), expressed in mm and defined as a circular area around the spot of the antibiotic or bioactive compound where the bacteria colonies do not grow [72]. The higher susceptibility of bacteria or fungi is associated with higher values of IZ and lower MIC values. These indicators were used in this study to compare the antibacterial and antifungal activity of Caucasian medicinal plants. In particular, the anticancer, anti-inflammatory, antibacterial, and antifungal properties of Caucasian medicinal plants were demonstrated [73]. The WHO has identified the discovery of novel antibacterial drugs to combat multidrug-resistant ESKAPE pathogens (*Enterococcus faecium*, *Staphylococcus aureus*, *Klebsiella pneumoniae*, *Acinetobacter baumannii*, *Pseudomonas aeruginosa*, and *Enterobacter* species) as a top priority. These pathogens, known for their ability to “escape” the effects of antimicrobial agents, are responsible for a significant portion of nosocomial infections [74]. Representatives of 15 families of Caucasian medicinal plant extracts have been explored for their efficacy against these pathogens (Table 1; the floristic regions are indicated in accordance with Figure 1). The most significant antimicrobial activity, based on MIC values, against resistant ESKAPE pathogens was revealed for the essential oil obtained from *Artemisia fragrans* Willd. stems [33], while acetone extract of *Hypericum alpestre* Steven. aerial part [36], aqueous extracts of *Juglans regia* L. green husks [41], acetone extract of *Sanguisorba officinalis* L. aerial part [36], methanol extract of *Peganum harmala* L. plant [66], and methanol [35] and ethanol extracts of *Agrimonia eupatoria* L. plants [36] showed significant activity against *S. aureus*. Ethanol extracts from *Peganum harmala* L. seeds and *Mentha pulegium* L. leaves demonstrated high activity against *K. pneumoniae* [45], whereas methanol extracts of *Acer cappadocicum* Gled. branches exhibited activity against *A. baumannii* [65].

The antimicrobial activity of Caucasian medicinal plants against *P. aeruginosa* was also investigated in many studies [28,30,33,34,36,41,49,50,54]. In particular, significant activity against *P. aeruginosa* was revealed for the essential oil obtained from *A. fragrans* flowers [33], the acetone extracts of *H. alpestre* aerial part [36] and *Lilium monadelphum* subsp. *armenum* (Miscz. ex Grossh.) Kudrjasch. bulb [36], as well as for the methanol extract of *Cyclamen coum* Mill. aerial parts [54], the acetone extracts of *A. eupatoria* plants [36], the ethanol extract of *Rosa canina* L. fruits [62], and the acetone extract of *S. officinalis* aerial parts [36]. In addition to their antimicrobial properties, medicinal plants have long been regarded as a traditional source of antifungal remedies [75]. Many studies demonstrated antifungal activities of Caucasian medicinal plants against different fungal species, including *Candida* [25,29,36,39,41,46,47,49,50,54,56,63,66]. Significant activity against the *Candida* species was revealed for the essential oil obtained from the aerial parts of *Clinopodium nepeta* (L.) Kuntze [43] and the acetone extracts of *A. eupatoria* plants [36], as well as the methanol extracts of *P. harmala* [66] and the essential oil obtained from the flowering aerial parts of *M. pulegium* [46]. Antifungal activities were also observed against *Aspergillus* sp., *Microsporum* sp., and *T. mentagrophytes* for the essential oil obtained from the aerial parts of *C. nepeta* [43]. Interestingly, essential oils obtained from the flowering aerial part of *M. pulegium* possessed activity against *A. niger* [46]. Remarkably, the growth of *Fusarium* and *Bipolaris* sp. was inhibited by an essential oil obtained from *Laurus nobilis* L. leaves [52]. Antifungal activity against *Penicillium* sp. was also revealed for acetone extracts of *A. eupatoria* aerial part [58]. Methanol extracts of the aerial parts and roots of *Filipendula ulmaria* (L.) Maxim. exhibited antifungal activity against a variety of species such as *A. alternata*, *C. albicans*, *T. longibrachiatum*, *P. cyclopium*, *P. canescens*, *F. oxysporum*, *A. niger*, *A. glaucus*, *T. harzianum*, *D. stemonitis*, and *P. fastigiate* [61].

In vivo studies of the antibacterial activity of Caucasian medicinal plants are very limited. Thus, in vivo studies demonstrated that a 3% *Laurus nobilis* L. leaf-supplemented diet in ulcerative colitis rat models increased the population of *Bifidobacteria* and *Lactobacillus* and significantly inhibited the growth of *Clostridium* and sulfate-reducing bacteria [76]. A total alkaloid extract of *P. harmala* at a concentration of 300 μg/mL decreased brown rot symptoms on potato tubers caused by aerobic, Gram-negative *R. solanacearum* in vivo [77]. It also was shown that a mixture of *Carum copticum* (L.) Benth. & Hook.f. ex Hiern essential oil and *P. harmala* extract encapsulated in chitosan nanoparticles is a superior treatment for the inhibition of fungi *A. alternata* in both in vitro and in vivo conditions [78]. The healing of infected wounds was promoted by gels prepared from *M. pulegium* essential oil loaded into nanostructured lipid carriers by increasing antibacterial properties [79]. The efficiency of *Allium sativum* L. extract against *S. flexneri* [26] and *P. aeruginosa* [80] was demonstrated in in vivo studies.

## 3. Major Groups of Antimicrobial Compounds of Caucasian Medicinal Plants

### 3.1. Phenolic Compounds

Phenolic compounds are secondary metabolites widely found in all higher plants, which play crucial roles in defense against plant diseases and herbivorous animal aggression [81]. Phenolic compounds are usually divided into simple phenolic compounds, polyphenols, and other phenolic compounds [82]. Simple phenolic compounds include simple phenolics (hydroxyphenols or dihydroxybenzenes) and phenolic acids (hydroxybenzoic and hydroxycinnamic acids, coumarins). Polyphenols include flavonoids, tannins, and stilbenes. Lignans and lignins are classified as other phenolic compounds [82]. Antibacterial, antitoxin, antiviral, and antifungal properties of phenolic compounds were described [83], with the best investigated antibacterial mechanism of polyphenols consisting of their direct binding to the bacterial cell membranes with consequent damage to them. Since Gram-positive bacteria do not possess outer membranes and have peptidoglycans on their surface, they seem to be the most susceptible to phenolic chemicals [84]. Phenolic compounds also have an impact on bacterial DNA structure, morphology, synthesis, and regulation [85]. They can transform pro-oxidants, and the oxidation of polyphenolic substances can lead to the production of hydrogen peroxide, causing DNA breaks [86]. Another mechanism underlying the antibacterial action of phenolic compounds is the inhibition of enzymatic activity [87]. This inhibition occurs through interactions between polyphenols and protein SH groups, as well as through non-specific interactions, contributing to the mechanism of enzyme inhibition [88].

#### 3.1.1. Phenolic Acids

**Scheme 1:**
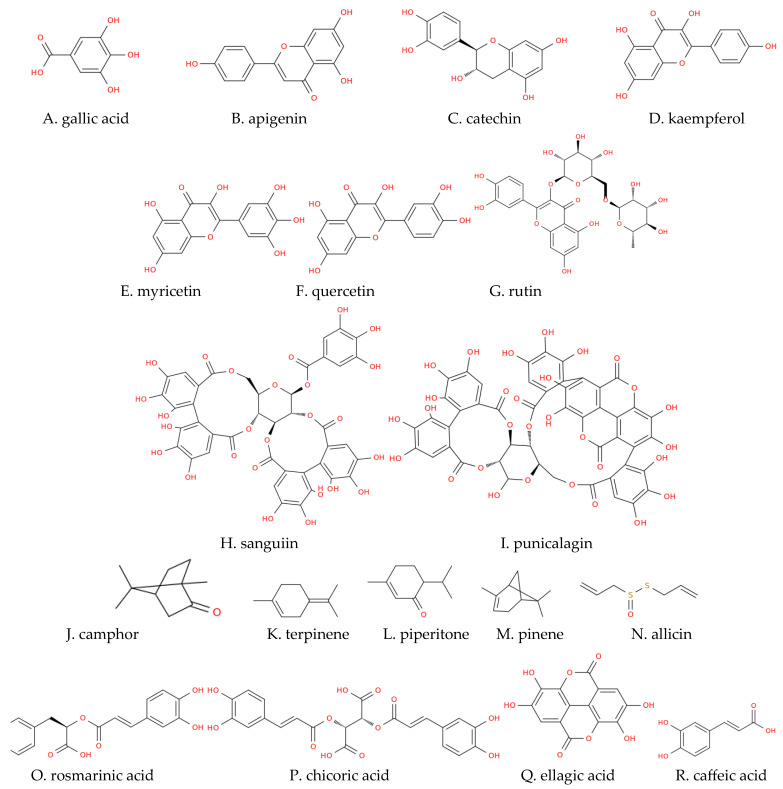

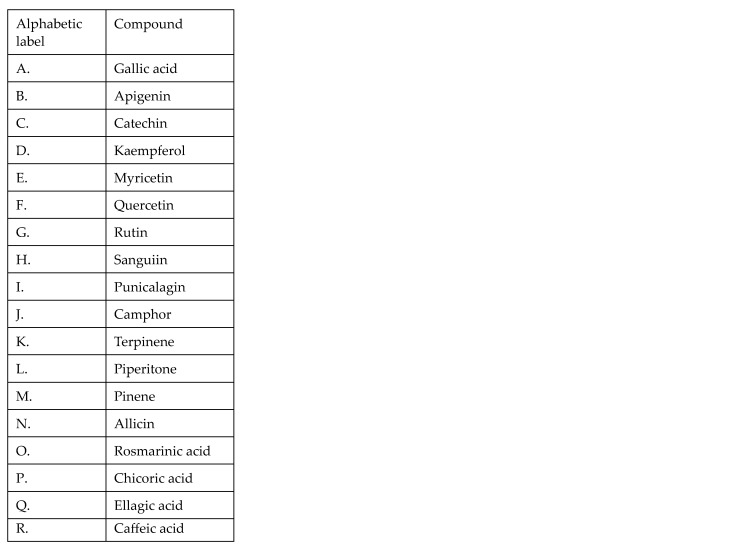
Molecular representation of some of the phytochemicals detected in Georgian flora in the order they are mentioned within this work. A list of compound names with their corresponding alphabetic labels is also provided.

Several representatives of different families of Caucasian medicinal plants considered in this study contained phenolic acids (Table 2). Gallic acid (Scheme 1) was the most commonly detected phenolic acid, found in the bulbs, flowers, and shoots of *G. transcaucasicus* [28], the bark of *J. regia* [41], aerial part and roots of *F. ulmaria* [61], and *S. officinalis* roots [89]. Gallic acid was the dominant phenolic acid in bulbs of *G. transcaucasicus* [28], as well as flowers and middle stems of *F. ulmaria* [60] (Table 2).

The proposed mechanism for the action of phenolic acids involves hyperacidification, affecting cell membrane potential and the operation of the sodium–potassium pump. Gram-positive bacteria are particularly susceptible to this antibacterial mechanism due to the absence of an outer membrane [84]. Additionally, phenolic acids such as gallic acid may disrupt the membrane of Gram-negative bacteria by chelating divalent cations [90]. In their undissociated form, phenolic acids can penetrate the cell membrane through passive diffusion, leading to cytosolic acidification and subsequent protein denaturation [84]. The antibacterial activity of ferulic and gallic acids was tested on *E. coli*, *P. aeruginosa*, *S. aureus*, and *L. monocytogenes*. For the studied bacteria, an MIC ranging from 100 to 1250 µg/mL was established for ferulic acid, whereas gallic acid acted with an MIC ranging from 500 to 2000 µg/mL [91]. It was concluded that gallic and ferulic acid reduce the negative surface charge, leading to local membrane rupture or pore formation and consequent leakage of essential intracellular constituents [91]. *p*-Coumaric acid was found in various parts of several Caucasian medicinal plants, including the bulbs of *A. sativum* and *A. ursinum* [25], leaves of *J. regia* [92], *L. nobilis* [49], leaves, flowers, and fruits of *F. ulmaria* [60], and the aerial part of *S. officinalis* [89]. Investigations into the antibacterial activity mechanism of *p*-coumaric acid unveiled a dual mechanism: it disrupts bacterial cell membranes and can bind to the phosphate anion in the DNA double helix, thereby intercalating into the DNA groove and affecting replication, transcription, and expression [93]. Phenolic acids have demonstrated in vitro and in vivo activity against *Candida* species [94], suggesting that, akin to the antibacterial effect, the antifungal effect may be linked to the impairment of cell surface hydrophobicity and charge [94]. Additionally, the impact of caffeic acid derivatives on 1,3-β-glucan synthase has been observed in the cell wall of *C. albicans* [95].

#### 3.1.2. Flavonoids

Different subclasses of flavonoids were detected in extracts of Caucasian medicinal plants (Table 2). Flavonoids exhibit multiple targets within bacterial cells, with their interaction with the cell membrane being the most extensively investigated mechanism [84]. Flavonoids with higher hydrophobicity can partition into the hydrophobic core of membranes. At the same time, those with greater hydrophilicity interact via hydrogen bonding with the polar head groups at the lipid–water interface, altering the physical and chemical properties of the membrane [96]. For instance, naringenin and sophoraflavanone were shown to reduce the fluidity of both the outer and inner membrane layers of bacterial cells [97]. The predominant group of flavonoids identified in Caucasian medicinal plants was flavanols (flavan-3-ols), encompassing apigenin, catechin and its derivatives, kaempferol, myricetin, quercetin, and rutin [98] (Scheme 1). The presence of apigenin was demonstrated in *G. transcaucasicus* flowers [28], *L. nobilis* leaves [49], and in *A. eupatoria* plants [99]. Catechin and its derivatives were detected in *F. ulmaria* leaves and roots [60], as well as in leaves, roots, and flowers of *S. officinalis* [64] and the fruits of *R. canina* [100]. Catechin and its derivatives compose the major fraction of flavonoids in flowers of *S. officinalis* [64]. Kaempferol was found in bulbs and was a major flavonoid in shoots of *G. transcaucasicus* [28] and *L. nobilis* leaves [49]; a derivative of kaempferol was found in *S. officinalis* leaves [64]. Quercetin and its derivatives were detected in *G. transcaucasicus* flowers and shoots [28], *L. nobilis* leaves [49], the aerial part of *F. ulmaria* [61], *R. canina* fruits [100], and in leaves and roots of *S. officinalis* [64]. Quercetin was the major flavonoid in *G. transcaucasicus* flowers [28]. On the other hand, rutin was revealed in *G. transcaucasicus* bulbs [28], *F. ulmaria* leaves, flowers, roots, and fruits [60], and *R. canina* fruits [100], whereas *L. nobilis* leaves contained myricetin [49], and it was also present in *R. canina* fruits [100]. Significant antibacterial activity of catechins against both Gram-positive and Gram-negative bacteria has been demonstrated, attributed to their ability to impair membrane integrity or induce membrane fusion [101,102,103]. This ability is associated with a high amount of phenolic hydroxyl groups, determining the high affinity of catechins for lipids, proteins, and nucleic acids [104]. Quercetin, apigenin, and sakuranetin were shown to possess a competitive inhibitory effect against bacterial enzymes involved in the type II fatty acid biosynthesis pathway: β-hydroxyacyl-acyl carrier protein dehydratase and β hydroxyacyl-ACP dehydrase [105]. It was suggested that inhibiting these enzymes impairs the lipid homeostasis of bacteria during both growth and stationary phases [106]. Myricetin inhibited the growth of Gram-positive and Gram-negative bacterial species via the inhibition of DNA helicase RepA and ATPase activities [107]. Rutin eradicated *P. aeruginosa* via a reduction in the production of protease, pyocyanin, rhamnolipid, and elastase and the disruption of membrane integrity [108]. Similarly, the bacteriostatic effect of kaempferol was mainly associated with the disruption of cellular integrity and leakage of cell contents [109]. The high antifungal activity of flavonoids against a wide range of pathogenic organisms was reviewed in [110]. In general, the antifungal action of flavonoids was associated with different mechanisms. Similarly to the case of their antibacterial action, these mechanisms involved plasma membrane disruption [111] and the effect of flavonoids on RNA and proteins [110]. However, it was demonstrated that flavonoids also induce mitochondrial dysfunction. Thus, the treatment with the flavone wogonin induced the accumulation of reactive oxygen species (ROS) in mitochondria and caused decreased membrane potential, as well as a reduction in ATP synthesis, and eventually, the contraction or cracking of fungal filaments [112]. Honey flavonoid extracts also inhibited cell division, preventing the proliferation of *C. albicans* phenotypes [113]. The inhibition of efflux-mediated pumps by many flavones (apigenin, chrysin, baicalein, luteolin, 6-hydroxyflavone, etc.), leading to fungi cell death, was demonstrated [114,115].

#### 3.1.3. Tannins

Tannins were detected in many species of Caucasian plants including *J. regia* leaves [92], *A. rosea plants* [53], *R. obtusifolius* seeds [99], *A. eupatoria* plants [99], the aerial part of *S. officinalis* [99], and the leaves, roots, and flowers of *S. officinalis* [64] (Table 2). *A. rosea* plants also contained phlobatannins [53], another class of ring-isomerized condensed tannins [116]. In studies where tannins were identified, mainly hydrolyzed tannins such as sanguiin and punicalagin gallate (Scheme 1) were revealed in Caucasian plants (Table 2). Hydrolyzable tannins have a polyhydric alcohol at their core, with the hydroxyl groups partially, or fully, esterified with either gallic or hexahydroxydiphenic acid [117]. The number of hydroxyl groups in tannins was used as a marker for assessing the antibacterial properties, positively associated with antioxidant characteristics [118]. Tannins inhibit the growth of diverse microbes, such as Gram-positive and Gram-negative bacteria, fungi, and yeasts [119]. For the majority of tannins, a bacteriostatic effect rather than bactericidal activity was revealed [120]. Tannins inhibited bacterial growth using different action mechanisms including iron chelation [119] and the inhibition of cell wall synthesis via inactivation of enzymes involved in cell wall synthesis or by binding to the cell wall [121]. Tannins also alter the structure of the bacterial membranes in *S. aureus*, increasing fluidity and disrupting the formation of virulent membrane vesicles, thus acting as potential enhancers of antibiotics [122]. Moreover, tannins affect the integrity and permeability of the cell wall and membrane by raising intracellular Ca^2+^ concentrations and affecting the activities of alkaline phosphatase and Na^+^/K^+^-ATPase [123], and tannin-loaded nanoparticles and hydrogels have demonstrated profound antibacterial effects [124]. Additionally, the antifungal effects of hydrolysable tannins were demonstrated against filamentous fungi and opportunistic yeasts [125], with the proposed mechanism of action involving the disruption of the cell wall and the plasma membrane, inducing leakage of the intracellular contents, such as sugars, as was demonstrated for the cell wall of *P. digitatum* [126].

### 3.2. Terpenes

Terpenes represent a large group of hydrocarbons consisting of five-carbon isoprene (C_5_H_8_) units as their basic building block. The modified family of terpenes are terpenoids, synthesized by the addition/removal of functional groups to/from terpenes [127] and comprising 40,000 compounds including terpenoid-derived indole alkaloids [128]. Terpenes and terpenoids were detected in the aerial part and the roots of *E. caucasicum* [30,31], *A. fragrans* aerial parts [32], roots [34] and flowers [33], and *P. fraxinifolia* stems [42], as well as in aerial parts of *C. nepeta* [43,44], *M. pulegium* [46,47], and *T. caucasicus* [48] and the leaves of *L. nobilis* [50]. In these Caucasian medicinal plants, camphor, terpinene, piperitone, pinene (Scheme 1), and their derivatives were the most frequently detected terpenes (Table 2). The antimicrobial and antifungal activity of terpenes and terpenoids was demonstrated and many studies were reviewed in [129]. Thus, a nanogel containing camphor and thymol provided complete growth suppression against *P. aeruginosa* and *S. aureus* [130]. Additionally, antibacterial properties of terpinen-4-ol against *E. faecalis*, *P. gingivalis*, *P. intermedia*, and *F. nucleatum* were demonstrated [131]. Piperitenone epoxide exhibited high antimicrobial efficiency against 28 strains of *S. aureus* and 10 strains of *E. coli* [132]. Antimicrobial and antifungal effects of α- and β-pinene were reported in many studies and reviewed in [133]. The proposed mechanism of antibacterial action of this class of compounds is the disruption of the bacterial membrane via the induction of oxidative stress and the impairment of several pathways involved in the bacterial membrane repair system, as was demonstrated in the study on *K. pneumoniae* [134]. The antifungal activity of terpenes against *C. albicans*, *S. aureus*, and *P. aeruginosa* was also demonstrated [135,136], with the proposed antifungal mechanism being the activation of specific downstream signaling pathways, which leads to the activation of genes involved in alternate metabolic and energy pathways. Moreover, drug efflux was prominently upregulated by terpenes which also caused the repression of genes mediating ribosome biogenesis and RNA metabolism [137].

### 3.3. Saponins

Saponins are natural compounds containing one or more sugar (glycon) and non-sugar parts (aglycon) connected via glycosidic bonds [138]. Due to the glycosylation of the hydrophobic aglycones, they can act as biological detergents and, when agitated in water, form foams (like in soap), which gave rise to the name of this group of compounds [139]. Saponins were found in aerial parts of *H. alpestre* [99], *C. coum* [55], *A. rosea* [53], *A. eupatoria* [99], *A. cappadocicum* [65], *P. harmala* plants [140], and *R. obtusifolius* seeds [99]. The biological properties such as antibacterial, anti-inflammatory, antifungal, and antiviral activities of various saponins were demonstrated [141,142], with the antibacterial and antifungal properties of saponins being associated with their detergent-like properties, providing increased permeability of bacterial cell membranes [138].

### 3.4. Alkaloids

Alkaloids are a large and structurally diverse group of natural products, detected in about 300 plant families [143]. Based on their chemical structure, alkaloids can be divided into three different types: true alkaloids (heterocyclics), protoalkaloids (non-heterocyclics), and pseudoalkaloids [144]. Among the investigated Caucasian plants, bioactive alkaloids were revealed in bulbs of *G. transcaucasicus* (native to Armenia, Azerbaijan, and Georgia) [29], *A. rosea* [53], *A. cappadocicum* [65], and *P. harmala* [66] plants. However, the specific types of alkaloids were not identified. Alkaloids inhibit bacterial growth through a variety of mechanisms; the most well-studied mechanism involves the inhibition of bacterial nucleic acid and protein synthesis [145]. Thus, the inhibitory action of berberine on DNA replication, RNA transcription, and protein biosynthesis in bacteria was shown [145]. Remarkably, sanguinarine inhibited the assembly of filamentous temperature-sensitive protein Z (FtsZ), essential in the process of bacterial cell division [146]. Moreover, alkaloids affect cell membrane permeability, causing damage to the cell membrane and cell wall [145,147]. Another mechanism of the antibacterial action of alkaloids is the inhibition of the efflux pump. This mechanism was demonstrated in the study of the effect of alkaloids extracted from *Callistemon citrinus* Skeels on *P. aeruginosa* [148]. Alkaloids are also capable of inhibiting various pathways of the bacterial metabolism. Thus, berberine increased the conversion and uptake of carbohydrates and decreased carbohydrate consumption by *S. pyogenes* [149]. A biological alkaline solution of *Aconitum carmichaeli* var. *carmichaeli* inhibited the metabolism of *S. aureus* [145]. At least 70 different plant-derived alkaloids were reviewed in [150] and were shown to possess significant antifungal activity in vitro. Similarly to the mechanism of antibacterial action, the antifungal action of alkaloids was related to membrane permeabilization, as well as to the inhibition of DNA, RNA, and protein synthesis [151].

### 3.5. Sulfur-Containing Compounds of Allium Species

Extracts of medicinal plants of the Alliaceae family found in the Caucasus area (including *A. atroviolaceum* and *A. ursinum*) were characterized by a high content of sulfur-containing compounds with proven antimicrobial, antioxidant, or antitumor activity [23,25,152]. Amongst the sulfur-containing compounds revealed in various Allium species discussed here, the most well-known and well-investigated compound is allicin (diallyl thiosulphate, Scheme 1), the major sulfur-containing compound in bulbs of *A. sativum* bulbs [153]. Antifungal and antimicrobial activities of allicin have been well known for a long time [153,154]. The antimicrobial and antifungal activity of allicin has been associated with its action as the inhibitor of sulfhydryl-dependent enzymes. This suggestion was supported by the finding that inhibition was alleviated or reduced by cysteine and glutathione [152,155]. The sulfur-containing compound ajoene from *A. sativum* caused a significant clearing of *P. aeruginosa* in a pulmonary infection model in mice in vivo [156]. Diallyl sulfide and diallyl disulfide isolated from *A. sativum* inhibited methicillin-resistant *S. aureus* infection in diabetic mice [157].

In summary, the investigation into major groups of antimicrobial compounds derived from Caucasian medicinal plants reveals a rich diversity of bioactive substances with significant potential for therapeutic applications. Phenolic compounds, including phenolic acids, flavonoids, and tannins, were prominently identified and exhibited multifaceted mechanisms of antimicrobial action. Phenolic acids, such as gallic acid and *p*-coumaric acid, exhibited antibacterial effects through membrane disruption and enzyme inhibition. Flavonoids, such as quercetin and rutin, showed antibacterial and antifungal properties by altering membrane integrity and interfering with bacterial enzymes. Tannins, particularly hydrolyzable tannins like sanguiin and punicalagin, exerted bacteriostatic effects and disrupted bacterial membranes and cell walls. Terpenes and terpenoids, abundant in many Caucasian medicinal plants, displayed antimicrobial and antifungal activities by disrupting bacterial membranes and inducing oxidative stress. Saponins, another group of natural compounds found in several plants, exhibited antibacterial properties attributed to their detergent-like effects, increasing bacterial cell membrane permeability. Alkaloids, identified in various plant species, inhibited bacterial nucleic acid and protein synthesis, damaged cell membranes, and inhibited efflux pumps, thereby exerting antimicrobial effects. Sulfur-containing compounds, particularly allicin from Allium species, demonstrated potent antimicrobial and antifungal activities by inhibiting sulfhydryl-dependent enzymes. Overall, the diverse array of antimicrobial compounds present in Caucasian medicinal plants offers promising avenues for the development of novel antimicrobial agents to combat infectious diseases. In conclusion, while Section 3 provides a comprehensive overview of the major groups of antimicrobial compounds found in Caucasian medicinal plants, it is essential to acknowledge some limitations and areas for further exploration. One notable gap is the need for more extensive studies elucidating the specific mechanisms of action of these compounds against various pathogens. While many studies have demonstrated the antimicrobial efficacy of phenolic compounds, terpenes, saponins, alkaloids, and sulfur-containing compounds, there remains a lack of clarity regarding their precise modes of action and potential synergistic effects. Additionally, further research is needed to optimize extraction methods to maximize the yield of bioactive compounds from medicinal plants while minimizing environmental impact. Moreover, the pharmacokinetics and bioavailability of these compounds in vivo warrant investigation to assess their potential as therapeutic agents for treating infectious diseases. Overall, continued interdisciplinary research efforts are necessary to unlock the full therapeutic potential of antimicrobial compounds derived from Caucasian medicinal plants and address the pressing global challenge of antibiotic resistance.

**Table 2:** Bioactive compounds with antimicrobial and antifungal activity revealed in Caucasian medicinal plants (susceptible microorganisms are shown in Table 1). Quantitative data on compound contents are shown when available.

Family	Plant Species	Plant Part	Extract Type	Class of Bioactive Compound	Bioactive Compound	Susceptible Microorganisms	References
Alliaceae	*A. atroviolaceum*	Bulb	essential oil	Sulfur-containing compounds	methyl methyl thiomethyl disulfide (61%) dimethyl trisulfide (15%) methyl allyl disulfide (4%)	Gram-positive	[158]
	*A. sativum*		aqueous extract	Simple phenolic compounds (phenolic acids)	gentisic acid 38 μg/g FW chlorogenic acid 36 μg/g FW 4-hydroxybenzoic acid 16 μg/g FW *p*-coumaric acid 26 μg/g FW	Gram-positive, Gram-negative, fungi	[25,156,157]
				Sulfur-containing compounds	alliin 1580 38 μg/g FW allicin 280 μg/g FW ajoene diallyl sulfide diallyl disulfide		
	*A. ursinum*		aqueous extract	Simple phenolic compounds (phenolic acids)	chlorogenic acid 40 μg/g FW *p*-coumaric acid 102 μg/g FW	Gram-positive, Gram-negative, fungi	[25]
				Sulfur-containing compounds	alliin 260 μg/g FW allicin 130 μg/g FW		
Amaryllidaceae	*G. transcaucasicus*	Bulb	99% methanol extract	Polyphenols (flavonoids)	naringin kaempferol rutin	Gram-positive, Gram-negative, fungi	[28]
				Simple phenolic compounds (phenolic acids)	gallic acid 439.5 μg/g DW syringic acid 117.7 μg/g DW ferulic acid 244.2 μg/g DW		
		Bulb	96% ethanol extract	Alkaloids Sterols Cardiac glycosides		Gram-positive, Gram-negative, fungi	[29]
		Flower	99% methanol extract	Polyphenols (flavonoids)	naringin 72.6 μg/g DW quercetin 915.5 μg/g DW apigenin 67.1 μg/g DW genistein 131.5 μg/g DW	Gram-positive, Gram-negative	[28]
				Simple phenolic compounds (phenolic acids)	gallic acid 112.1 μg/g DW syringic acid 926.2 μg/g DW		
		Shoot	99% methanol extract	Polyphenols (flavonoids)	naringin 112.9 μg/g DW quercetin 259.3 μg/g DW kaempferol 411.5 μg/g DW genistein 202 μg/g DW	Gram-positive, Gram-negative	[28]
				Simple phenolic compounds (phenolic acids)	gallic acid 345.8 μg/g DW syringic acid 705.5 μg/g DW ferulic acid 412 μg/g DW		
Apiaceae	*E. caucasicum*	Roots	essential oil	Fatty Acid Esters	hexyl isovalerate hexyl valerate	Gram-positive, Gram-negative	[30]
				Terpenes	trans-pinocarvyl acetate		
		Aerial part		Fatty Acid Esters	hexyl isovalerate		[31]
				Terpenes	trans-pinocarvyl acetate caryophyllene oxide		
		Shoots	80% methanol extract	Simple phenolic compounds (phenolic acids)	rosmarinic acid chicoric acid (Scheme 1)		[159]
Asteraceae	*A. fragrans*	Aerial part	essential oil	Terpenes	α-thujone β-thujone 1,8-cineole davanone d camphor cadinol verbenene ortho-oci-men	Gram-positive, Gram-negative	[32]
		Leaves	essential oil	Terpenes	Chrysanthenon 23.8% 1,8-cineole 23.7% β-caryophyllene 9.6% *p*-cymene 7.7% filifolide-A 5.7% filifolone 5.7% camphor terpinene-4-ol artemisyl acetate camphene	Gram-positive, Gram-negative	[33,34]
		Roots	essential oil	Terpenes	camphor 67% camphene 16.9%	Gram-positive, Gram-negative	[34]
		Stem	essential oil	Terpenes	camphor 1,8-cineole borneol artedouglasia oxide a chrysanthenyl acetate	Gram-positive, Gram-negative	[33]
		Flowers	essential oil	Terpenes	camphor 1,8-cineole terpinene-4-ol borneol carvacrol	Gram-positive, Gram-negative	[33]
Hypericaceae	*H. alpestre*	Aerial part	99% methanol extract	Saponins Steroids Polyphenols	Flavonoids Coumarins	Gram-positive, Gram-negative, fungi	[99]
Juglandaceae	*J. regia*	Leaves	80% methanol extract	Polyphenols (flavonoids)	-	Gram-positive, Gram-negative	[38]
			99% methanol extract	Polyphenols (tannins) Antioxidants	alpha-tocopherol	Gram-positive, Gram-negative	[92]
				Simple phenolic compounds (phenolic acids)	caffeic acid *p*-coumaric acid ellagic acid malic acid chlorogenic acid		
		Bark	99% methanol extract	Simple phenolic compounds (phenolic acids)	chlorogenic acid caffeic acid ferulic acid sinapic acid gallic acid ellagic acid vanillic acid	Gram-positive, Gram-negative, fungi	[41]
		Green husks	99% methanol extract	Simple phenolic compounds (phenolic acids)	coumaric acid ellagic acid chlorogenic acid	Gram-positive, Gram-negative, fungi	[41]
	*P. fraxinifolia*	Stem	essential oil	Terpenes Fatty Acids	2,4-heptadienal hexanol 2-pyrrolidinone menthone menthol thymol vinylguajacol hexadecanoic acid	Gram-positive, Gram-negative, fungi	[42]
Lamiaceae	*C. nepeta*	Aerial part	essential oil	Terpenes	piperitenone oxide 47.8% limonene 18.6% piperitone oxide 13.6% 6-hydroxycarvotanacetone 5.1%	Gram-positive, Gram-negative, fungi	[43,44]
	*M. pulegium*	Flowering aerial part	essential oil	Terpenes	piperitone 38% piperitenone 33% α-terpineol 4.7% 1,8-cineole menthone 4% borneol 3% pulegone 0.6%	Gram-positive, Gram-negative, fungi	[46,47]
	*T. caucasicus*	Aerial part	essential oil	Terpenes	1,8-cineol 21.5% thymol 12.6% β-fenchyl alcohol 8.7% nerolidol 7.8% terpinolene 7.2% *α*-pinene 7% myrcene 6.8%	Gram-positive, Gram-negative, fungi	[48]
Lauraceae	*L. nobilis*	Leaves	essential oil	Simple phenolic compounds (phenolic acids)	chlorogenic acid 48.1 μg/g DW caffeic acid 789.3 μg/g DW *p*-coumaric acid 375. 9 μg/g DW sinapic acid 1513.9 μg/g DW ferulic acid 70.4 μg/g DW cinnamic acid 513.4 μg/g DW	Gram-positive, Gram-negative, fungi	[49,50]
				Simple phenolic compounds (phenolic acids)	protocatechuic acid 68.6 μg/g DW salicylic acid 29. 4 μg/g DW syringic acid 789.1 μg/g DW		
				Polyphenols (flavonoids)	myricetin 47.2 μg/g DW quercetin 44.9 μg/g DW kaempferol 688.1 μg/g DW luteolin 839.1 μg/g DW apigenin 262.7 μg/g DW hesperetin 31.2 μg/g DW		
				Terpenes	1,8-cineole 31.9% *a*-terpinyl acetate 5.9% β-pinene 2.5% sabinene 8.8% β-linalool 4.9% piperitenone isomenthone pulegone		[50,52,160]
Malvaceae	*A. rosea*	Whole plant	98% ethyl acetate extract	Saponins Phenolic compounds (tannins and phlobtannins) Terpenoids Alkaloids Cardiac glycosides	-	Gram-positive, Gram-negative	[53]
Polygonaceae	*R. obtusifolius*	Seeds	99% methanol extract	Saponins Terpenoids Phenolic compounds Coumarins	-	Gram-positive, Gram-negative, fungi	[99]
			99.8% acetone extract	Polyphenols (tannins and flavonoids)	-		
Primulacea	*C. coum*	Aerial part	99% methanol extract	Proteins Phenolic compounds Saponins Cardiac glycosides Steroids	-	Gram-positive, fungi	[55]
	*P. macrocalyx*	Whole plant	99% methanol extract	Polyphenols (flavonoids)	3′-methoxyflavone 2′-methoxyflavone 2′,5′-dimethoxyflavone 2′-methoxy-5′-hydroxyflavone 3′-hydroxy-4′,5′-dimethoxyflavone 5,6,2′,6′-tetramethoxyflavone 5,6,2′,3′,6′-pentamethoxyflavone 3′-hydroxyflavone 2′-hydroxyflavone 5,6,8,2′,6′-pentamethoxyflavone 5,6,2′-trimethoxyflavone	Fungi	[56]
Rosaceae	*A. eupatoria*	Whole plant	99% methanol extract	Saponins Steroids Polyphenols (tannins)	luteolin apigenin	Gram-positive, Gram-negative, fungi	[99]
			99.8% acetone extract	Steroids Phenolic compounds	-		
	*F. ulmaria*	Middle leaves	60% ethanol extract	Simple phenolic compounds (phenolic acids Polyphenols (flavonoids)	gallic acid 0.8 mg/g extract caftaric acid 0.6 mg/g extract chlorogenic acid 1.3 mg/g extract *p*-coumaric acid 0.2 mg/g extract catechin 4.1 mg/g extract rutin 4.8 mg/g extract isoquercitrin 2.6 mg/g extract ellagic acid 0.4 mg/g extract	Gram-positive, Gram-negative	[60]
		Middle stem	60% ethanol extract	Simple phenolic compounds (phenolic acids) Polyphenols (flavonoids)	gallic acid 1.3 mg/g extract caftaric acid 0.05 mg/g extract chlorogenic acid 0.2 mg/g extract ellagic acid 0.4 mg/g extract isoquercitrin 0.07 mg/g extract	Gram-positive, Gram-negative	[60]
		Flowers	60% ethanol extract	Simple phenolic compounds (phenolic acids) Polyphenols (flavonoids)	gallic acid 5.8 mg/g extract caftaric acid 2.9 mg/g extract chlorogenic acid 0.3 mg/g extract caffeic acid 0.1 mg/g extract *p*-coumaric acid 0.04 mg/g extract ellagic acid 5.8 mg/g extract rutin 4.2 mg/g extract isoquercitrin 2.4 mg/g extract spiraeoside 20.4 mg/g extract cymaroside 0.09 mg/g extract	Gram-positive, Gram-negative	[60]
		Fruits	60% ethanol extract	Simple phenolic compounds (phenolic acids) Polyphenols (flavonoids)	gallic acid 4.3 mg/g extract caftaric acid 1.5 mg/g extract chlorogenic acid 0.6 mg/g extract *p*-coumaric acid 0.7 mg/g extract caffeic acid 0.07 mg/g extract ellagic acid 3.4 mg/g extract rutin 2.1 mg/g extract spiaeoside 2.4 mg/g extract isoquercitrin 0.5 mg/g extract	Gram-positive, Gram-negative	[60]
		Aerial part	99% methanol extract	Simple phenolic compounds (phenolic acids) Polyphenols (flavonoids)	gallic acid 7.02 mg/g extract ellagic acid 8.9 mg/g extract rutin 6.2 mg/g extract quercetin 15. 5 mg/g extract catechin 11.3 mg/g extract	Gram-positive, Gram-negative, fungi	[61]
		Roots	60% ethanol extract	Simple phenolic compounds (phenolic acids) Polyphenols (flavonoids)	gallic acid 0.1 mg/g extract salicylic acid 0.6 mg/g extract ellagic acid 1.2 mg/g extract catechin 8.0 mg/g extract rutin 0.7 mg/g extract isoquercitrin 0.05 mg/g extract	Gram-positive, Gram-negative, fungi	[60]
		Roots	99% methanol extract	Polyphenols (flavonoids)	catechin 17.2 mg/g extract epicatechin 3.1 mg/g extract	Gram-positive, Gram-negative, fungi	[61]
	*R. canina*	Fruits	hexane/acetone/ethanol (2:1:1), and 0.05% (*w*/*v*) butylated hydroxytoluene extract	Carotenes	carotene lycopene	Gram-positive, Gram-negative, fungi	[63]
			Acetone/water (80:20 *v*/*v*) extract	Simple phenolic compounds (phenolic acids) Polyphenols (flavonoids)	vanilic acid 260 μg/kg DW cafeic acid 2 μg/kg DW syringic acid 110 μg/kg DW gallic 298 μg/kg DW ellagic acid 80 μg/kg DW procatechuic acid 210 μg/kg DW myricetin 5.4 μg/kg DW rutin 22 μg/kg DW catechin 11.9 μg/kg DW quercetin 1.5 μg/kg DW		[100]
	*S. officinalis*	Aerial part	99% methanol extract	Phenolic compounds (tannins, flavonoids and coumarins) Steroids Glycosides Quinones	-	Gram-positive, Gram-negative, fungi	[99]
			petroleum ether extract, followed by extraction with 80% methanol	Simple phenolic compounds (phenolic acids)	caffeic acid *p*-coumaric acid syringic acid vannilic acid ferulic acid		[89]
		Roots	70% ethanol extract	Polyphenols (tannins)	2,3-hexahydroxydiphenoyl-glucose 15.3 mg/g DW sanguiin H-10 derivative 9 mg/g DW punicalagin gallate 3.8 mg/g DW sanguiin H-1 7.7 mg/g DW galoyl-bis-hexahydroxydiphenyl-glucoside, isomer 2.8 mg/g DW ellagic acid 2.3 mg/g DW	Gram-positive, Gram-negative, fungi	[64]
				Polyphenols (flavonoids)	c-type (epi)catechin trimer 10.5 mg/g DW b-type (epi)catechin dimer catechin 17.2 mg/g DW		
				Simple phenolic compounds (phenolic acids)	3-caffeoylquinic acid caffeic acid-glucoside chlorogenic acid *p*-coumaroylquinic acid ellagic acid		
			petroleum ether extract, followed by extraction with 80% methanol	Simple phenolic compounds (phenolic acids)	gallic acid protocatechuic acid		[89]
		Leaves	70% ethanol extract	Polyphenols (tannins) Polyphenols (flavonoids) Simple phenolic compounds (phenolic acids)	2,3-hexahydroxydiphenoyl-glucose 6.9 mg/g DW sanguiin H-10 derivative 1.9 mg/g DW punicalagin gallate 9.9 mg/g DW sanguiin H-1 8.3 mg/g DW b-type (epi)catechin dimer 6.1 mg/g DW catechin 7.4 mg/g DW quercetin-galloyl-glucoside 2.9 mg/g DW quercetin-glucoside 18.6 mg/g DW kaempferol-glucuronide 7.6 mg/g DW 3-caffeoylquinic acid 1 mg/g DW caffeic acid-glucoside 2.4 mg/g DW chlorogenic acid 1.6 mg/g DW *p*-coumaroylquinic acid 2.8 mg/g DW	Gram-positive, Gram-negative	[64]
		Flowers	70% ethanol extract	Polyphenols (tannins) Polyphenols (flavonoids) Simple phenolic compounds (phenolic acids)	2,3-hexahydroxydiphenoyl-glucose 12.6 mg/g DW sanguiin H-10 derivative 8.3 mg/g DW punicalagin gallate 11.1 mg/g DW sanguiin 19.7 mg/g DW ellagic acid pentoside 2.6 mg/g DW cyanidin-glucoside 0.5 mg/g DW b-type (epi)catechin dimer 5 mg/g DW catechin 2.3 mg/g DW 3-caffeoylquinic acid 2.1 mg/g DW caffeic acid-glucoside 5.4 mg/g DW chlorogenic acid 2.2 mg/g DW *p*-coumaroylquinic acid 3.1 mg/g DW	Gram-positive, Gram-negative	[64]
Sapindaceae	*A. cappadocicum*	Whole plant	99% methanol extract	Alkaloids Saponins Flavone glycosides Quinones		Gram-positive, Gram-negative, fungi	[65]
Zygophyllaceae	*P. harmala*	Whole plant	99% methanol extract	Alkaloids Saponins Flavone glycosides	peganine harmaline	Gram-positive, Gram-negative, fungi	[66,140]

## 4. Mechanisms of Action of Antibacterial and Antifungal Plant Compounds

Bacterial infections pose an escalating threat to human health worldwide. While antibiotics have historically provided an effective means of treating such infections, the widespread and often excessive use of these drugs has led to the emergence and spread of antibiotic-resistant bacteria. As a consequence, antimicrobial resistance has become a pressing global concern, resulting in the loss of efficacy of many antibiotics, and, tragically, this phenomenon contributes to the deaths of at least 700,000 people annually across the globe [161]. The first reports of antibiotic resistance date back to the 1950s with *Salmonella*, *Shigella*, and *E. coli* species [162]. However, the comprehension of the mechanisms providing bacterial resistance took several decades [163]. In contrast, research into resistance against antifungal agents has progressed more slowly, likely due to the recognition of mycoses as a significant threat in the mid-1970s (as reviewed in [164]).

**Figure 2:**
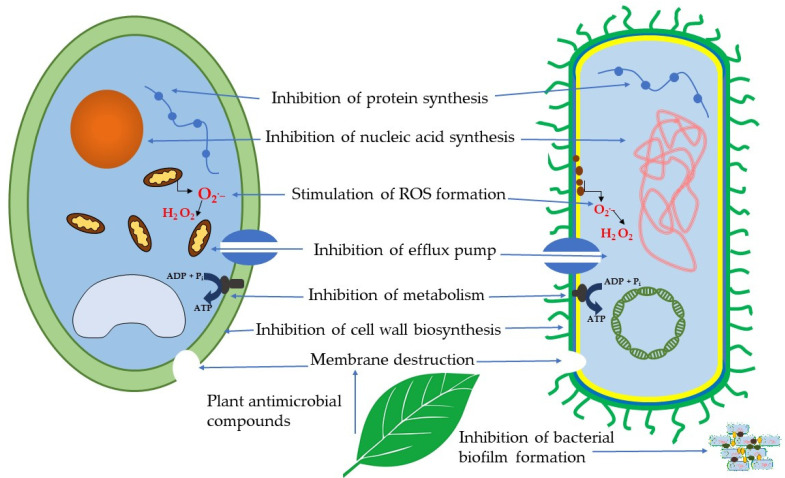
Mechanisms of action of antibacterial and antifungal plant compounds.

As was discussed above, plants produce a variety of secondary metabolites, such as phenolic compounds, terpenes, saponins, alkaloids, and sulfur-containing compounds. These compounds have been used since ancient times in folk medicine for the treatment of various diseases [73]. Since phytochemicals can act via different mechanisms or target sites different from those of antibiotics, their use either alone or in combination with antibiotics can be efficient for the suppression of bacterial resistance [11]. The current elucidated mechanisms of action of antibacterial and antifungal secondary metabolites as antimicrobial agents are related to a variety of targets in bacterial and fungal cells (Figure 2).

### 4.1. Membrane Destruction

Similar to certain antibiotics, like polymyxins, which induce structural alterations in bacterial membranes [165], plant-derived bioactive compounds are capable of causing the destruction of bacterial cell membranes. This ability has been reported for various classes of phenolic compounds [166]. For example, quinones bind to the adhesin complex on the cell wall, eventually leading to its lysis [167]. Experimental studies on the interaction between flavonoids and biomembranes have shown that flavonoids with a planar configuration, such as quercetin, rutin, and tiliroside, reduce the thickness and fluidity of lipid bilayers, thereby increasing membrane permeability [168]. Tannins have been shown to deactivate microbial adhesins, enzymes, and membrane transporter systems [169]. Terpenes, on the other hand, break down the outer membrane of various Gram-negative bacteria, causing the release of lipopolysaccharides [170]. Alkaloids derived from *Dicranostigma leptopodum* (Maxim.) Fedde altered the cell permeability of *K. pneumoniae,* exerting a significant antibacterial effect on its growth [145]. Terpenes, owing to their lipophilic properties, can traverse the phospholipid bilayer of bacteria, demonstrating antibacterial or bactericidal effects [171]. The efficacy of several terpenes (carvacrol, thymol, eugenol) against *S. typhimurium*, *E. coli*, *P. fluorescens*, *B. thermosphacta*, and *S. aureus* was examined, revealing membrane damage [172]. Flavonoids modified the cell membrane of *Candida* spp., enhancing cell permeability and inhibiting the function of plasma membrane proteins [173]. The alkaloid tomatidine, isolated from *Solanum tuberosum* L., inhibited two enzymes involved in ergosterol biosynthesis in *C. albicans*, *C. krusei*, and *S. cerevisiae* [174].

### 4.2. Inhibition of Cell Wall Biosynthesis

The bacterial cell wall, composed of a network of peptide and glycan strands that are covalently cross-linked, serves as a crucial target for antibacterial medications due to its ability to provide high mechanical strength and prevent osmotic lysis [175]. Inhibiting enzymes involved in fatty acid biosynthesis is a promising strategy for antimicrobial agents to impede bacterial growth. In this regard, various steps of fatty acid biosynthesis have been shown to be inhibited by flavonoids. For instance, quercetin, apigenin, and sakuranetin have been found to inhibit 3-hydroxyacyl-ACP dehydrase from *H. pylori* [176]. Additionally, different subclasses of flavonoids have been shown to inhibit 3-ketoacyl-ACP synthase and malonyl CoA-acyl carrier protein [177,178]. Quercetin and apigenin have also demonstrated inhibitory effects on peptidoglycan synthesis, an essential constituent of the bacterial cell wall [179]. Acidic terpenoids such as enfumafungin, ascosteroside, arundifungin, and ergokonin A, which exhibit antifungal activity against *Aspergillus* and *Candida*, are proposed to inhibit β(1,3)-D-glucan synthase, an enzyme involved in glucan synthesis, the primary polysaccharide in the fungal cell wall (reviewed in [180]). Studies have shown that these compounds selectively target β(1,3)-D-glucan synthases in a non-competitive manner [181]. Remarkably, glabridin, a flavonoid, exerts its antifungal mechanism through cell wall deformation [110].

### 4.3. Inhibition of Biofilm Formation

Biofilms represent well-organized communities of microbes entrenched within a self-produced extracellular matrix [182]. Within these biofilms, bacteria can endure harsh conditions and secrete various polymers such as polysaccharides, extracellular DNA (e-DNA), and amyloidogenic proteins [183]. Bacterial biofilm infections, whether stemming from pre-existing conditions, hospital-acquired infections, or the use of different medical devices, pose significant threats to public health [184]. In this respect, numerous studies have showcased the ability of flavonoids to inhibit bacterial biofilm formation [184]. For example, phloretin, a flavonoid belonging to the dihydrochalcone class, hindered the formation of fimbriae—elongated filamentous polymeric protein structures located on the surface of bacterial cells in *E. coli*—by reducing the expression of curli genes (csgA, csgB), ultimately impeding biofilm formation [182]. Flavonoids have also been observed to induce pseudo-multicellular aggregation of *S. aureus*, thereby inhibiting biofilm formation [185,186]. The alkaloid anguinarine significantly suppressed biofilm formation by *P. rettgeri* through the blockade of expression and formation of biofilm substances [187]. Similarly, the alkaloid berberine exhibited inhibition of biofilm formation in drug-resistant *E. coli* strains [188].

Flavonoids such as quercetin and apigenin were found to inhibit biofilm formation by *C. albicans*, while ellagic and caffeic acid from propolis were effective against the biofilms of *C. auris* (reviewed in [151]). Prenylflavanone 8PP demonstrated inhibition of *C. albicans* biofilms by increasing the production of ROS and reactive nitrogen intermediates [189].

### 4.4. Inhibition of Nucleic Acid and Protein Synthesis

The crucial bacterial enzyme responsible for catalyzing the ATP-dependent negative supercoiling of double-stranded closed-circular DNA is DNA gyrase [190]. In a study assessing the antibacterial activity of flavonoids against *E. coli*, the inhibitory effects of these compounds on DNA gyrase were evaluated through DNA supercoiling assays. Among the compounds investigated, kaempferol exhibited the most potent inhibitory activity [191]. A docking study aimed at identifying potential DNA gyrase B inhibitors from *C. difficile* suggested that several flavonoids have significantly higher binding affinity compared to the known inhibitor novobiocin [192]. Additionally, flavonoids such as luteolin and myricetin were found to inhibit DNA gyrase, a crucial replicative enzyme responsible for separating and/or rearranging DNA double strands in *E. coli* [193]. Various flavonoids have been proposed as potent inhibitors of several DNA and RNA polymerases, as well as viral reverse transcriptase [194]. The already mentioned alkaloid berberine demonstrated inhibition of DNA replication, RNA transcription, and protein biosynthesis in bacteria [145]. It was also observed to inhibit the assembly of FtsZ protein, crucial for bacterial cell cycle and division [146], and to inhibit the synthesis of proteins related to cell growth [145]. Catechin inhibited nucleic acid synthesis in *C. albicans*, reducing the expression of hypha-specific genes in the mitogen-activated protein kinase cascade and the cyclic adenosine 3,5-monophosphate pathway [195]. Similarly, several flavonoids inhibited the growth of *C. lunatus* by inhibiting nucleic acid synthesis [196]. Gallic acid extracted from *Paeonia rockii* (S. G. Haw & Lauener) T. Hong & J. J. Li ex D.Y. Hong inhibited protein synthesis in *C. albicans*, leading to a reduction in the number of hyphal cells and germ tubes [197].

### 4.5. Inhibition of Metabolism

Numerous studies have demonstrated the inhibition of various pathways of bacterial metabolism by bioactive plant compounds. For instance, the inhibitory effect of 17 bioflavonoid compounds on purified F1 or membrane-bound F1Fo of *E. coli* was evidenced [198]. Catechin and epigallocatechin gallate were shown to inhibit the enzymatic activity of F1Fo-ATPase and lactate dehydrogenase in *S. mutans* [199]. Flavonoids were found to hinder the growth of *P. aeruginosa* and *S. aureus* by affecting the enzymatic activity of ATPase [169]. The alkaloid berberine impacted the carbohydrate metabolism of *S. pyogenes* by upregulating ATP-binding cassette transporter and phosphotransferase systems involved in carbohydrate uptake and increasing carbohydrate conversion [149]. Additionally, various terpenes isolated from vegetal sources were tested on eleven fungi species, revealing their antifungal effect through the inhibition of respiration, succinate dehydrogenase, and NADH oxidase activities [200].

### 4.6. Stimulation of ROS Generation

The induction of high levels of reactive oxygen species through various methods has been extensively explored for the treatment of infections [201]. Numerous plant compounds have been demonstrated to augment ROS production. The inhibitory mechanism of catechin on the growth of both Gram-positive and Gram-negative bacteria has been linked to the induction of ROS production via membrane permeabilization, a phenomenon confirmed through the formation and treatment of bacterial liposomes [202]. For example, berberine has been shown to stimulate excessive ROS production in *S. pyogenes* [149]. The flavonoid bacalein has been found to induce ROS accumulation and subsequent cell apoptosis in *Candida* species [115]. Flavonoids found in honey [113] and quercetin have been observed to inhibit the growth of *C. albicans* by increasing intracellular ROS levels [203].

### 4.7. Inhibition of Efflux Pumps

Efflux pumps, found in most living cells, are transport proteins responsible for moving substrates from the cell to the external environment. They play a crucial role in detoxifying bacterial or fungal cells by removing accumulated drugs, and their high expression is often associated with drug resistance [204]. Consequently, inhibiting efflux pumps is essential for combating drug resistance. One of the earliest discovered efflux inhibitors of plant origin is the alkaloid reserpine, which has been demonstrated to effectively inhibit the Bmr (*Bacillus* multidrug resistance) efflux pump of *B. subtilis* [66]. Plant alkaloids such as ellagic and tannic acids have also shown inhibitory effects on efflux activities in *A. baumannii* [30]. Flavonoids from various subclasses have exhibited activity as bacterial efflux pump inhibitors (as reviewed in [205]). Numerous studies have demonstrated the inhibition of bacterial efflux pumps by terpenes [206], with *S. aureus* being the most commonly studied bacterium and carvacrol being the most investigated terpene. Importantly, efflux pumps of Gram-positive bacteria have been found to be more susceptible to the action of terpenes, which may involve either inhibition of gene expression or interaction with the binding site of membrane-associated efflux proteins [206]. The polyphenol curcumin has been shown to modulate efflux pump activity in *S. cerevisiae* [207]. Additionally, various flavonoids (such as apigenin, chrysin, baicalein, luteolin, tangeritin, scutellarein, 6-hydroxyflavone, and sedonan A) have been found to inhibit efflux-mediated pumps, leading to cell death in fungi (reviewed in [110]). In summary, a myriad of mechanisms of action for antibacterial and antifungal compounds derived from plants, including those found in Caucasus plants, have been uncovered, offering promising avenues for combating antimicrobial resistance. These mechanisms are multifaceted, involving various targets within bacterial and fungal cells, and encompass membrane destruction, inhibition of cell wall biosynthesis, inhibition of biofilm formation, inhibition of nucleic acid and protein synthesis, inhibition of metabolism, stimulation of ROS generation, and inhibition of efflux pumps. Plant-derived secondary metabolites, such as phenolic compounds, terpenes, saponins, alkaloids, and sulfur-containing compounds, have long been utilized in folk medicine for treating various ailments. Their diverse mechanisms of action or target sites, distinct from those of antibiotics, offer potential for combating bacterial resistance either alone or in combination with antibiotics. However, gaps persist in fully elucidating the mechanisms of action of antibacterial and antifungal secondary metabolites. Understanding these diverse mechanisms is crucial for developing effective strategies to combat antimicrobial resistance. Further research into the therapeutic potential of plant-derived antimicrobial compounds holds promise for addressing the growing threat of antimicrobial resistance and improving global public health outcomes.

## 5. Conclusions and Future Perspectives

In conclusion, this study underscores the significant antimicrobial potential of Caucasian medicinal plants, many of which are endemic species, such as *Eryngium caucasicum* and *Thymus caucasicum*. These plants have long been valued for their therapeutic properties, offering promising avenues for drug discovery against a wide range of pathogens. The diverse array of bioactive compounds present in these endemic species underscores their value as sources of novel therapeutic agents in the fight against infectious diseases, particularly in the face of rising antimicrobial resistance. Moreover, this study underscores the critical role of traditional knowledge in guiding modern drug discovery efforts. Indigenous communities have long recognized the medicinal properties of endemic plants and their sustainable use, providing valuable insights that can inform scientific research and conservation initiatives. However, it is crucial to address the challenges associated with standardizing methodologies for assessing antimicrobial activity, particularly when comparing results across different studies. Standardization efforts can facilitate more accurate comparisons and reliable data interpretation in future research. Looking ahead, the identified antimicrobial compounds from Caucasian medicinal plants, including endemic species, hold great promise for further investigation and development into novel therapeutic agents. Given the urgent global need for effective treatments against multidrug-resistant pathogens, these natural sources represent valuable reservoirs for drug discovery efforts. Continued research efforts should focus on elucidating the mechanisms of action of these bioactive compounds, optimizing extraction techniques, and conducting preclinical and clinical trials. Moreover, it is essential to prioritize conservation efforts aimed at protecting endemic species and their habitats, ensuring their availability for future generations and supporting ongoing research endeavors in natural-product-based drug discovery. By combining scientific innovation with the preservation of biodiversity and traditional knowledge associated with endemic plants, we can unlock the full therapeutic potential of Caucasian medicinal flora and contribute to addressing the global challenge of antimicrobial resistance.

## Figures and Tables

**Table 1:** Antimicrobial screening of Caucasian medicinal plants.

Family	Species	Distribution	Plant Part	Extract Type	Susceptible Microorganism						Reference
					Gram-Positive	MIC (mg/mL)/IZ, mm	Gram-Negative	MIC (mg/mL)/IZ, mm	Fungi	MIC (mg/mL)/IZ, mm	
Alliaceae	*Allium atroviolaceum* Hornem. Ex Steud.	Entire Caucasus	Bulbs	70% methanol extract	*S. mitis* *S. mutans* *S. sanguinis* *S. salivarius*	6.25/- 6.25/- 3.12/- 6.25/-	-	-	-	-	[23]
			Bulbs	aqueous extract	*B. subtilis* *B. cereus* *S. aureus*	0.31/42.6 1.25/25 0.63/19.4	*S. flexneri* *K. pneumoniae* *E. coli*	1.25/12 2.50/8.5 2.50/23.5	-	-	[24]
	*Allium sativum* L.	Entire Caucasus	Bulbs	70% ethanol extract aqueous extract	*S. aureus*	12.5/15	*E. coli* *S. flexneri* *P. aeruginosa*	6.25/14 5 */- 0.6/25	*C. albicans* *C. parapsilosis*	12.5/27 6.25/22	[25,26,27]
	*Allium ursinum* L.	Entire Caucasus	Bulbs	70% ethanol extract	*S. aureus*	25/16	*E. coli*	6.25/17	*C. albicans* *C. parapsilosis*	6.25/29 6.25/20	[25]
Amaryllidaceae	*Galanthus transcaucasicus* Fomin	Eastern and Southern Transcaucasia and Eastern Caucasus	Bulbs	99% methanol extract	*B. subtilis* *B. cereus* *S. aureus*	-/82 -/71 -/35	*E. coli* *P. aeruginosa*	-/85 -/46	-	-	[28]
			Flowers		*B. subtilis* *B. cereus* *S. aureus*	-/10 -/122 -/76	*E. coli* *P. aeruginosa*	-/116 -/98	-	-	[28]
			Shoots		*B. subtilis* *B. cereus* *S. aureus*	-/112 -/118 -/92	*E. coli* *P. aeruginosa*	-/129 -/106	-	-	[28]
			Bulbs	96% ethanol extract	*B. subtilis* *S. aureus*	9.28/- 1.17/-	-	-	*C. albicans*	150 U/mL *	[29]
Apiaceae	*Eryngium caucasicum* Trautv.	Entire Caucasus	Roots	essential oil	*S. aureus* *B. subtilis.*	0.5/19 0.5/18	*E. coli* *P. aeruginosa*	0.50/7 >1/9	-	-	[30]
			Aerial part		*S. aureus* *B. subtilis*	0.5/19 0.4/18	*E. coli* *P. aeruginosa*	0.85/7 >1/9	-	-	[31]
Asteraceae	*Artemisia fragrans* *Willd.*	South-Western Transcaucasia, Southern Transcaucasia, Eastern Transcaucasia	Aerial part	essential oil	*S. aureus* *S. epidermidis* *B. subtilis*	ni/12 ni/11 124.8 × 10^−6^/23	*E. coli* *K. pneumoniae* *P. vulgaris*	ni/16.3 3.9 × 10^−6^/18.3 ni/21.3	-	-	[32]
			Leaves	c	*S. aureus* *E. faecalis* *B. anthracis* *S. epidermidis* *S. saprophyticus*	-/9 -/10 3.10/25 0.006/20 0.03/10	*E. coli* *P. aeruginosa* *S. flexneri* *S. paratyphi* *S. typhi*	-/8 -/ni 0.0125/15 0.0125/15 0.025/10	-	-	[33,34]
			Roots	essential oil	*S. aureus* *E. faecalis*	-/12 -/14	*E. coli* *P. aeruginosa*	-/9 -/ni	-	-	[34]
			Stem	essential oil	*B. anthracis* *E. faecalis* *S. aureus* *S. epidermidis* *S. saprophyticus*	ni/ni 0.013/15 0.006/20 0.03/10 ni/ni	*E. coli* *P. aeruginosa*	0.025/10 ni/ni	-	-	[33]
			Flowers	essential oil	*B. anthracis* *E. faecalis* *S. aureus* *S. epidermidis* *S. saprophyticus*	ni/ni 0.006/20 0.003/25 0.006/20 25/10	*E. coli* *P. aeruginosa*	0.025/10 0.025/10	-	-	[33]
Hypericaceae	*Hypericum alpestre* Steven.	Entire Caucasus	Aerial part	99% methanol extract	*S. aureus*	0.26/-	*E. coli*	1.02/-	-	-	[35]
			Aerial part	99.8% acetone extract	*S. aureus* *B. subtilis*	0.13/12 0.13/10	*E. coli* *P. aeruginosa* *S. typhimurium*	1.02/ni 0.06/21 0.51/ni	*C. albicans* *C. guilliermondii*	1.02/ni 1.02/ni	[36]
Juglandaceae	*Juglans regia* L.	Entire Caucasus	Leaves	99% methanol extract	-	-	*Klebsiella* sp. *E. coli*	-/15.3 -/21	-	-	[37]
			Leaves	80% methanol extract	*S. mutans* *S. salivarius* *S. sanguinis* *A. viscosus*	125/- 15.6/- 15.6/- 187.50/-	-	-	-	-	[38]
			Bark	ethyl acetate extract	-	-	-	-	*C. albicans* *C. dubliniensis* *C. glabrata* *P. guilliermondii*	-/18.3 -/14.3 -/10.3 -/12.3	[39]
				80% methanol extract	*S. aureus* *Streptococcus* spp.	-/18.4 -/12/0	*E. coli* *K. pneumoniae* *P. multocida* *M. haemolytica*	-/ni -/ni -/17.1 -/16.6	-	-	[40]
			Green husk	aqueous extract	*B. cereus* *B. subtilis* *S. aureus*	0.1/>9 10/5 0.1/>9	*E. coli* *P. aeruginosa K. pneumoniae*	100/ni 100/5 100/ni	*C. albicans* *C. neoformans*	100/ni- 100/ni-	[41]
	*Pterocarya fraxinifolia* (Poir.) Spach	Entire Caucasus	Leaves	99% methanol extract			*Klebsiella* sp. *E. coli*	-/16 -/21.7	-	-	[37]
			Stem	essential oil	*S. epidermidis* *S. aureus* *B. subtilis*	-/27 -/10 -/10	*S. paratyphi* *S. dysenteriae* *P. vulgaris* *E. coli* *K. pneumoniae*	-/ni -/ni -/ni -/ni -/ni	*C. albicans* *A. niger*	-/ni -/11	[42]
Lamiaceae	*Clinopodium nepeta* (L.) Kuntze	Western Transcaucasia	Aerial part	essential oil	*B. cereus* *S. sanguinis*	2.50/- 2.50/-	*E. coli * *P. aeruginosa* *S. typhimurium*	5/- 10/- 1.25/-	*A. flavus* *A. terreus* *C. albicans* *M. canis* *M. gypseum* *T. mentagrophytes*	2/- 0.4/- 2/- 0.4/- 0.4/- 0.4/-	[43,44]
	*Mentha* *pulegium* L.	Western and Eastern Ciscaucasia, Western Transcaucasia, South-Western Transcaucasia, Southern Transcaucasia, Eastern Transcaucasia	Leaves	48% ethanol extract	*S. pneumoniae*	0.11/17.2	*K. pneumoniae*	0.23/8.1	-	-	[45]
			Flowering aerial part	essential oil	*S. aureus* *S. epidermidis* *B. cereus* *L. monocytogenes* *E. faecalis*	0.5 */21 1 */19 1 */16 1 */8 4 **/10.7	*V. cholera * *E. coli* *S. typhimurium*	0.5 */13 4 */ni 2 */ni	*A. niger* *C. albicans* *C. tropicalis*	0.25 */10 1 */16 16 **/18.6	[46,47]
	*Thymus caucasicus* Willd. ex Benth.	Western Transcaucasia, Central Transcaucasia, Eastern Transcaucasia	Aerial part	essential oil	*S. faecalis* *S. typhi* *S. aureus*	0.005/- ni/- 0.1/-	*E. coli*	0.005/-	*C. albicans*	ni/-	[48]
Lauraceae	*Laurus nobilis* L.	Entire Caucasus	Leaves	essential oil	*B. cereus* *E. faecalis* *E. faecium* *S. aureus* *L. monocytogenes*	-/12 -/11.5 -/12 -/12.5 -/12.0	*S. abony* *P. vulgaris* *K. pneumoniae* *Shigella flexneri* *P. aeruginosa* *P. fluorescens* *P. mirabilis*	-/9.5 -/8.5 ni ni ni ni ni	*C. albicans* *C. glabrata* *C. tropicalis* *A. niger* *A. versicolor* *P. citrinum* *P. expansum*	-/16.3 -/10 -/12 -/6 -/7.7 -/5.7 -/9.7	[49,50]
				70% ethanol extract	-	-	*E. coli* *S. typhi*	-/11.3 -/14.5	-	-	[51]
				99.8% acetone extract	*E. faecalis* *S. pneumoniae* *S. aureus*	0.13/24.0 0.13/37.2 0.25/16.7	*E. coli* *P. mirabilis*	0.25/23.3 0.13/24.0	*F. solani* *F. oxysporum* *A. alternata* *Bipolaris* sp.	0.25/- 4/- 32/- 0.5/-	[52]
Liliaceae	*Lilium monadelphum* subsp. *armenum* (Miscz. ex Grossh.) Kudrjasch.	South-Western Transcaucasia, Southern Transcaucasia	Bulbs	99.8% acetone extract	*S. aureus* *B. subtilis*	0.51/10 0.51/9	*E. coli* *P. aeruginosa* *S. typhimurium*	0.51/9 0.13/11 0.51/9	*C. albicans* *C. guilliermondii*	ni/ni ni/ni	[36]
Malvaceae	*Alcea rosea* L.	Eastern Caucasus, Eastern Transcaucasia	Flowers	48% ethanol extract	*S. pneumoniae*	375/6.9	*K. pneumoniae*	680/3.2	-	-	[45]
			Whole plant	98% ethyl acetate extract	*S. aureus*	-/25	*P. vulgaris* *K. pneumoniae* *E. coli* *P. aeruginosa*	-/13 -/18 -/28 -/20	-	-	[53]
Polygonaceae	*Rumex obtusifolius* L.	Entire Caucasus	Leaf	99.8% acetone extract	*S. aureus* *B. subtilis*	-/11 -/ni	*E. coli* *P. aeruginosa* *S. typhimurium*	-/ni -/12 -/10	*C. albicans* *C. guilliermondii*	-/ni -/12	[36]
			Root	99.8% acetone extract	*S. aureus* *B. subtilis*	-/10 -/9	*E. coli* *P. aeruginosa* *S. typhimurium*	-/ni 64/12 512/ni	*C. albicans* *C. guilliermondii*	-/9 -/13	[36]
			Inflorescence	99.8% acetone extract	*S. aureus* *B. subtilis*	-/11 -/11	*E. coli* *P. aeruginosa* *S. typhimurium*	-/10 -/9 512/ni	*C. albicans* *C. guilliermondii*	-/ni -/9	[36]
			Seeds	99.8% acetone extract	*S. aureus* *B. subtilis*	0.256/12 0.128/10	*E. coli* *P. aeruginosa* *S. typhimurium*	512/10 128/10 512/12	*C. albicans* *C. guilliermondii*	ni/ni ni/10	[36]
Primulacea	*Cyclamen coum* Mill.	Eastern Transcaucasia	Bulb	99% ethanol extract	*E. faecalis* *B. subtilis* *S. epidermidis* *S. aureus*	-/8 -/ni -/ni -/ni	*E. coli* *P. aeruginosa* *S. typhimurium*	-/ni -/ni -/ni	*C. albicans*	-/8	[54]
			Leaf	99% ethanol extract	*E. faecalis* *B. subtilis* *S. epidermidis* *S. aureus*	-/ni -/ni -/ni -/ni	*E. coli* *P. aeruginosa* *S. typhimurium*	-/ni -/ni -/ni	*C. albicans*	-/ni	[54]
			Flower	99% ethanol extract	*B. subtilis* *E. faecalis* *S. epidermidis* *S. aureus*	-/ni -/ni -/ni -/ni	*E. coli* *P. aeruginosa* *S. typhimurium*	-/ni -/ni -/ni	*C. albicans*	-/11	[54]
			Aerial part	99% methanol extract	*S. aureus*	0.13/-	*E. coli* *P. aeruginosa*	0.013/- 0.006/-	-	-	[55]
	*Primula macrocalyx* Bunge	Western Transcaucasia, Central Transcaucasia, Eastern Transcaucasia, South-Western Transcaucasia, Southern Transcaucasia	Entire plant	99% methanol extract	-	-	-	-	*C. rugosa*	-/20	[56]
Rosaceae	*Agrimonia eupatoria* L.	Entire Caucasus	Whole plant	99% methanol extract	*S. aureus*	0.26/-	*E. coli*	0.51/-	-	-	[35]
				99.8% acetone extract	*S. aureus* *B. subtilis*	0.26/10 0.13/11	*E. coli* *P. aeruginosa* *S. typhimurium*	0.51/10 0.13/11 0.51/10	*C. albicans* *C. guilliermondii*	0.51/9 0.26/12	[36]
			Seeds	99% methanol extract	*B. cereus* *B. subtilis*	0.75/- 0.75/-	-	-	-	-	[57]
			Aerial part	99.8% acetone extract	*B. cereus* *E. faecalis*	0.31/- 2.50/-	*P. mirabilis * *K. pneumoniae * *S. enterica * *S. typhimurium*	2.5/- 10/- 10/- 10/-	*P. italicum * *P. chrysogenum * *A. flavus * *A. niger*	2.50/- 2.50/- 10/- 20/-	[58]
				75% methanol extract	*S. aureus*	+	-	-	-	-	[59]
	*Filipendula ulmaria* (L.) Maxim.	Western Transcaucasia, Central Transcaucasia, Eastern Transcaucasia	Leaves	60% ethanol extract	*B. subtilis*	-/ni	*P. aeruginosa*	-/10.7	-	-	[60]
			Stem	60% ethanol extract	*B. subtilis*	-/ni	*P. aeruginosa*	-/6.9	-	-	[60]
			Flowers	60% ethanol extract	*B. subtilis*	-/ni	*P. aeruginosa*	-/10.7	-	-	[60]
			Fruits	60% ethanol extract	*B. subtilis*	-/13.1	*P. aeruginosa*	-/13.3	-	-	[60]
			Roots	60% ethanol extract	*B. subtilis*	-/ni	*P. aeruginosa*	-/11.1	-	-	[60]
			Aerial part	99% methanol extract	*E. faecalis*	0.31/-	*E. coli* *P. aeruginosa K. pneumoniae*	0.16/- 5/- 5/-	*C. albicans* *T. harzianum* *T. longibrachiatum* *P. cyclopium* *P. canescens* *A. niger* *A. glaucus* *F. oxysporum* *A. alternata* *D. stemonitis* *P. fastigiata*	>10/- 2.5/- 5/- 2.5/- 10/- >10/- 5/- 2.5/- >10/- >10/- 5/-	[61]
			Roots	99% methanol extract	*E. faecalis*	0.31/-	*E. coli * *P. aeruginosa K. pneumoniae*	0.63/- 5/- 5/-	*C. albicans* *T. harzianum* *T. longibrachiatum* *P. cyclopium* *P. canescens* *A. niger* *A. glaucus* *F. oxysporum* *A. alternata* *D. stemonitis* *P. fastigiata*	>10/- 10/- 10/- 5/- >10/- >10/- >10/- 2.5/- >10/- >10/- 10/-	
	*Rosa canina* L.	Entire Caucasus	Fruits	96% ethanol extract	*B. cereus* *C. perfringens* *L. monocytogenes*	0.13/8.33 0.51/4.00 0.26/6.00	*E. coli* *K. oxytoca* *P. aeruginosa*	0.03/14.33 0.06/12.00 0.13/9.67	-	-	[62]
			Fruit	hexane/acetone/ethanol (2:1:1), 0.05% butylated hydroxytoluene extract	*S. aureus* *E. faecalis* *B. cereus*	4/- 4/- 4/-	*E. coli* *S. enteritidis* *E. aerogenes * *P. aeruginosa*	4/- 4/- 4/- 2/-	*C. albicans*	2/-	[63]
	*Sanguisorba officinalis* L.	Entire Caucasus	Aerial part	99% methanol extract	*S. aureus*	0.56/-	*E. coli*	1.02/-	-	-	[35]
				99.8% acetone extract	*S. aureus* *B. subtilis*	0.13/13 0.13/13	*E. coli* *P. aeruginosa* *S. typhimurium*	0.256/102 0.064/12 0.256/11	*C. albicans* *C. guilliermondii*	0.51/10 0.51/10	[36]
			Roots	99.8% acetone extract	*S. aureus* *B. subtilis*	-/10 -/10	*E. coli* *P. aeruginosa* *S. typhimurium*	-/10 -/10 -/10	*C. albicans* *C. guilliermondii*	-/10 -/10	[36]
			Roots	70% ethanol extract	*S. aureus*	-/5.50	*E. coli* *P. aeruginosa*	-/6.56 -/6.55	-	-	[64]
			Leaves		*S. aureus*	-/8.43	*E. coli* *P. aeruginosa*	-/11.46 -/11.33	-	-	
			Flowers		*S. aureus*	-/2.5	*E. coli* *P. aeruginosa*	-/2.66 -/4.60	-	-	
Sapindaceae	*Acer cappadocicum* Gled.	Entire Caucasus	Branches with leaves	99% methanol extract	*B. subtilis*	-/16.5	*E. coli* *K. pneumoniae* *S. enterica* *A. baumannii*	-/16.3 -/16.5 -/15 -/11	*F. fujikuroi * *R. oryzae* *P. ultimum*	-/48% *** /52% *** /49% ***	[65]
Zygophyllaceae	*Peganum harmala* L.	Western Transcaucasia, Central Transcaucasia, Eastern Transcaucasia, South-Western Transcaucasia, Southern Transcaucasia	Seeds	48% ethanol extract	*S. pneumoniae*	0.08/27.2	*K. pneumoniae*	0.15/10.1	-	-	[45]
			Whole plant	99% methanol extract	*S. aureus*	0.15/-	*E. coli * *P. aeruginosa*	5/- 1.5/-	*C. albicans*	0.063/-	[66]

IZ—inhibitory zone, mm; MIC—minimal inhibitory concentration, mg/mL; for IZ, the highest value is shown; for MIC, the lowest value is shown; +—inhibition was detected, but MIC and IZ were not determined; - was not determined; ni—no inhibition was detected. * MIC was expressed as μL/mL. ** MIC was expressed as %, *v*/*v*, determined by resazurin microtiter assay. *** Percentage of inhibition of mycelial growth is shown.

## Abbreviations

DW	dry weight
FW	fresh weight
Bacterial species	
*A. alternata*	*Alternaria alternata*
*A. baumannii*	*Acinetobacter baumannii*
*A. flavus*	*Aspergillus flavus*
*A. niger*	*Aspergillus niger*
*A. terreus*	*Aspergillus terreus*
*A. viscosus*	*Actinomyces viscosus*
*A. versicolor*	*Aspergillus versicolor*
*B. anthracis*	*Bacillus anthracis*
*B. cereus*	*Bacillus cereus*
*B. subtilis*	*Bacillus subtilis*
*B.* *thermosphacta*	*Brochothrix thermosphacta*
*C. albicans*	*Candida albicans*
*C. auris*	*Candida auris*
*C. difficile*	*Clostridium difficile*
*C. dubliniensis*	*Candida dubliniensis*
*C. glabrata*	*Candida glabrata*
*C. guilliermondii*	*Candida guilliermondii*
*C. krusei*	*Candida krusei*
*C. lunatus*	*Cochliobolus lunatus*
*C. neoformans*	*Cryptococcus neoformans*
*C. parapsilosis*	*Candida parapsilosis*
*C. perfringens*	*Clostridium perfringens*
*C. rugosa*	*Candida rugosa*
*C. tropicalis*	*Candida tropicalis*
*D.* *stemonitis*	*Doratomyces* *stemonitis*
*E. aerogenes*	*Enterobacter aerogenes*
*E. coli*	*Escherichia coli*
*E. faecalis*	*Enterococcus faecalis*
*E. faecium*	*Enterococcus faecium*
*F. fujikuroi*	*Fusarium fujikuroi*
*F. nucleatum*	*Fusobacterium nucleatum*
*F. oxysporum*	*Fusarium oxysporum*
*F. solani*	*Fusarium solani*
*H. pylori*	*Helicobacter pylori*
*K. oxytoca*	*Klebsiella oxytoca*
*K. pneumoniae*	*Klebsiella pneumoniae*
*L. monocytogenes*	*Listeria monocytogenes*
*M. canis*	*Microsporum canis*
*M. gypseum*	*Microsporum gypseum*
*M. haemolytica*	*Mannheimia haemolytica*
*P. aeruginosa*	*Pseudomonas aeruginosa*
*P.* *canescens*	*Penicillium* *canescens*
*P. chrysogenum*	*Penicillium chrysogenum*
*P. citrinum*	*Penicillium citrinum*
*P.* *cyclopium*	*Penicillium* *cyclopium*
*P. digitatum*	*Penicillium digitatum*
*P. expansum*	*Penicillium expansum*
*P. fastigiate*	*Phialophora fastigiate*
*P. fluorescens*	*Pseudomonas fluorescens*
*P. gingivalis*	*Porphyromonas gingivalis*
*P. guilliermondii*	*Pichia guilliermondii*
*P. italicum*	*Penicillium italicum*
*P. intermedia*	*Prevotella intermedia*
*P. mirabilis*	*Proteus mirabilis*
*P. multocida*	*Pasteurella multocida*
*P. rettgeri*	*Providencia rettgeri*
*P. ultimum*	*Pythium ultimum*
*P. vulgaris*	*Proteus vulgaris*
*R. solanacearum*	*Ralstonia solanacearum*
*R. oryzae*	*Rhizopus oryzae*
*S. abony*	*Salmonella abony*
*S. aureus*	*Staphylococcus aureus*
*S. cerevisiae*	*Saccharomyces cerevisiae*
*S. dysenteriae*	*Shigella dysenteriae*
*S. enterica*	*Salmonella enterica*
*S. enteritidis*	*Salmonella enteritidis*
*S. epidermidis*	*Staphylococcus epidermidis*
*S. flexneri*	*Shigella flexneri*
*S. mitis*	*Streptococcus mitis*
*S. mutans*	*Streptococcus mutans*
*S. paratyphi*	*Salmonella paratyphi*
*S. pneumoniae*	*Streptococcus pneumoniae*
*S. pyogenes*	*Streptococcus pyogenes*
*S. salivarius*	*Streptococcus salivarius*
*S. sanguinis*	*Streptococcus sanguinis*
*S. saprophyticus*	*Staphylococcus saprophyticus*
*S. typhi*	*Salmonella typhi*
*S. typhimurium*	*Salmonella typhimurium*
*T.* *harzianum*	*Trichoderma* *harzianum*
*T.* *longibrachiatum*	*Trichoderma* *longibrachiatum*
*V. cholera*	*Vibrio cholera*
Plant species	
*Agrimonia eupatoria* L.	*A. eupatoria*
*Acer cappadocicum* Gled.	*A. cappadocicum*
*Aconitum carmichaeli* var. *carmichaeli*	*A. carmichaeli*
*Alcea rosea* L.	*A. rosea*
*Allium atroviolaceum* Hornem. Ex Steud.	*A. atroviolaceum*
*Allium sativum* L.	*A. sativum*
*Allium ursinum* L.	*A. ursinum*
*Artemisia fragrans Willd.*	*A. fragrans*
*Carum copticum* (L.) Benth. & Hook.f. ex Hiern	*C. copticum*
*Clinopodium nepeta* (L.) Kuntze	*C. nepeta*
*Cyclamen coum* Mill.	*C. coum*
*Eryngium caucasicum* Trautv.	*E. caucasicum*
*Filipendula ulmaria* (L.) Maxim.	*F. ulmaria*
*Galanthus transcaucasicus* Fomin	*G. transcaucasicus*
*Hypericum alpestre* Steven.	*H. alpestre*
*Juglans regia* L.	*J. regia*
*Laurus nobilis* L.	*L. nobilis*
*Lilium monadelphum* subsp. *armenum* (Miscz. ex Grossh.) Kudrjasch.	*L*. *armenum*
*Mentha pulegium* L.	*M. pulegium*
*Peganum harmala* L.	*P. harmala*
*Primula macrocalyx* Bunge	*P. macrocalyx*
*Pterocarya fraxinifolia* (Poir.) Spach	*P. fraxinifolia*
*Rosa canina* L.	*R. canina*
*Rumex obtusifolius* L.	*R. obtusifolius*
*Sanguisorba officinalis* L.	*S. officinalis*
*Thymus caucasicus* Willd. ex Benth.	*T. caucasicus*
